# Predicting Errors, Violations, and Safety Participation Behavior at Nuclear Power Plants

**DOI:** 10.3390/ijerph17155613

**Published:** 2020-08-04

**Authors:** Tingru Zhang, Zhaopeng Liu, Shiwen Zheng, Xingda Qu, Da Tao

**Affiliations:** 1 State Key Laboratory of Nuclear Power Safety Monitoring Technology and Equipment, China Nuclear Power Engineering Co., Ltd., Shenzhen 518172, China; zhangtr@szu.edu.cn (T.Z.); liuzhaopeng@cgnpc.com.cn (Z.L.); 2 Institute of Human Factors and Ergonomics, College of Mechatronics and Control Engineering, Shenzhen University, Shenzhen 518060, China; 1800292007@email.szu.edu.cn (S.Z.); quxd@szu.edu.cn (X.Q.)

**Keywords:** errors, violations, safety participation, organization and planning, nuclear power plant safety

## Abstract

Commissioning workers at nuclear power plants have long been ignored in previous studies, although their performance is closely related to the overall safety of plants. This study aimed to explain and predict three types of behavior, i.e., errors, violations, and safety participation, of commissioning workers, under the general framework of the theory of planned behavior (TPB) and by considering organization and planning factors. The validity of the model was evaluated with a sample of 167 commissioning workers who completed a self-reported questionnaire. The results showed that perceived behavioral control, along with organization and planning, significantly affected all types of behavior. It was also found that violations and errors were a direct result of attitude. Besides, errors were predicted by subjective norm; unexpectedly, this occurred in a positive way. These findings revealed the underlying mechanisms for the development of errors, violations, and safety participation among commissioning workers and provided practical implications for safety improvement at the commissioning workplace.

## 1. Introduction

The burning of fossil fuels emits large quantities of air pollutants that are harmful to the environment and public health [1,2]. To cope with these adverse impacts, many countries have tried to reduce their reliance on fossil fuels by developing clean and renewable energy, such as nuclear, wind, solar, and geothermal energy. Among these emerging energy sources, nuclear energy has gained much attention for its relatively low cost and stable production rate [3]. By 2017, there were about 450 nuclear power reactors worldwide, providing over 10% of the world’s electricity [4]. These numbers will keep increasing, as many new nuclear power plants are on order or planned [4], notably in Asian countries such as China. China recently has restarted the construction of nuclear power plants after a two-year hiatus [5], with about 15 under construction and more about to start construction. With the intense development, commissioning, one of the key phases prior to the start of nuclear power plants’ commercial operation, has raised much attention recently.

According to the International Atomic Energy Agency [6], commissioning is “the process by means of which systems and components of facilities and activities, having been constructed, are made operational and verified to be in accordance with the design and to have met the required performance criteria”. Commissioning is a safety-critical phase in the nuclear power plant lifecycle because it aims at noticing and fixing all deficiencies and possible errors before the plant commences operation. Great challenges lie in commissioning phase, such as the pressure of completing work within a limited time, the need to test new features of the plants, and the possible unexpected emergencies with regard to the fuel loading stage [7]. Incidents or accidents during commissioning can lead to consequences ranging from minor (e.g., malfunction of devices) to severe (e.g., prolonged completion time and even excessive radiation leakage). Much evidence in occupational safety literature showed that the aberrant behavior of workers, such as errors and violations, was one important cause of accidents [8,9], while workers’ safety participation behavior significantly reduced accident risk [10,11]. These findings indicate that the behavioral performance of the commissioning workers is of great importance to the safety and efficiency of commissioning phase and to the future safety of nuclear power plants when they are in operation.

Numerous studies have been conducted and many theories have been proposed to explain individuals’ aberrant and safety participation behavior [12]. The investigated worker groups include construction workers [13], aviation maintainers [12], and oilrig workers [14]. Workers at nuclear power plants have also attracted some research attention, but the focus has been mostly on control room operators [15,16]. No research has focused on the behavior of commissioning workers. There is a great difference between commissioning workers and control room operators in terms of core tasks and ways of organization. For instance, the main tasks at control room are to monitor the status of nuclear power units and handle abnormities based on specific regulations. The main commissioning tasks, in contrast, are to start up the nuclear power units and conduct experiments to examine if the units can run normally, and such tasks are filled with uncertainties [7]. Another significant difference is that the control room usually has stable team members, while commissioning tends to build flexible teams based on specific tasks [17]. The complex and challenging features of commissioning may make the behavioral characteristics of commissioning workers quite different from those of control room operators. Therefore, studies focusing on commissioning workers are needed to understand the mechanisms behind behavior during commissioning.

Another research gap is that most of the available studies mentioned above emphasized only the aberrant behaviors and ignored workers’ intent and enthusiasm for proactively engaging in safety-promoting behavior. Such voluntary behavior, often referred to as safety participation, extends beyond compliance with safety regulations and is not formally rewarded by the organization but can be beneficial to the overall safety of the organization [18,19]. To fill these gaps, this study aimed to propose models to predict and explain errors, violations, and safety participation behavior of commissioning workers. Related findings not only shed theoretical light on understanding antecedents of commissioning workers’ behavior, but also provide guidelines to policymakers when designing countermeasures to advocate or reduce certain types of behavior. The remainder of the introduction describes the theoretical foundation underlying this study and the model and hypotheses proposed.

### 1.1. Errors, Violations, and Safety Participation Behavior

Aberrant behavior, which refers to a straying from the path [20], could be classified into two types based on whether the behavior is intentional or not. Instances of intentional aberrant behavior are traditionally referred to as violations, which involve deliberate deviation from rules or practices that are important in maintaining the safety of a particular task or job [12]. This is opposed to errors, which refer to unintended outcomes caused by slips, lapses, and mistakes made by individuals [12,20]. Both types of behavior are shaped by cognitive, psychological, and social factors. However, the effect sizes of these factors on violations and errors are different; the former are more closely associated with social and psychological factors such as attitude, while the latter are more strongly affected by deficiencies in cognitive abilities such as information processing efficiency and organization skills [21]. They also differ in their associations with demographic variables such as age and gender [22] and in their contributions to accidents [23,24].

While compliance with safety rules and regulations is important in lowering the risk of accidents, organizations also need individuals’ proactive participation in safety [18]. Safety participation refers to an employee’s voluntary participation in safety activities that is beyond the employee’s formal role but does contribute to the development of a supportive safety environment [25]. Examples of safety participation include promoting safety programs within the workplace, helping coworkers, and raising safety concerns [26,27]. Safety participation has been reported to be a significant predictor of occupational accidents [10,28], the effect size of which is even greater than that of aberrant behavior [27].

### 1.2. Theory of Planned Behavior

Many theories have been proposed and applied to explain human behavior [29]. For instance, theory of self-efficacy posits that two types of expectancies, i.e., outcome expectancy and self-efficacy expectancy, exert powerful influence on individual behavior [30]. Self-determination theory, by contrast, suggests that motivations are the strongest determinants in shaping who we are and how we behave. In particular, individuals are motivated to engage in certain activities when their needs for competence, connection, and autonomy are fulfilled [31]. Another well-recognized theory that tries to explain human behavior from a social cognitive perspective is the theory of planned behavior (TPB) proposed by Ajzen [32]. It is derived from the original theory of reasoned action (TRA) [33]. TRA was developed based on the premise that individuals make reasoned decisions to engage in specific behavior by evaluating the information available to them. It proposes that two factors, attitude and subjective norm, directly determine an individual’s behavioral intention. Attitude refers to the degree to which a person has a favorable or unfavorable evaluation or appraisal of the behavior in question, while subjective norm refers to the perceived social pressure to perform or not to perform the behavior [33]. The formed intention further decides whether the individual would actually do that behavior.

Although intention is a strong predictor of actual behavior, there are times when individuals do not execute an action, despite having the intention to act in a certain manner, because of external factors that fall outside their control. As a result, TRA is limited to only predicting behavior of people who have complete volitional control [34]. To extend the explanatory scope, TPB was proposed; this theory incorporates perceived behavioral control, defined as the perceived ease or difficulty of performing the behavior [32], into the model. TPB posits that attitude, subjective norm, and perceived behavioral control together shape one’s intention to engage in certain behavior, and this intention, together with perceived behavioral control, determine the probability that an actual action will be taken. As a general rule, the more favorable the attitude and subjective norm with respect to the behavior are, the greater the perceived behavioral control is, and the stronger an individual’s intention to perform the behavior should be [32]. Recent evidence suggested that perceived behavioral control and subjective norm could also impact behavior indirectly by influencing attitude [12,35,36].

As a type of intentional or planned behavior, violations at workplace should follow the framework in TPB. This has been confirmed by some empirical studies that reported the effectiveness of TPB in explaining violations in aviation maintenance [12], construction worksites [37], and road transportation [38,39,40]. For instance, Fogarty and Shaw [12] reported that a positive attitude towards violations and the perception that other people in work commit violations (i.e., subjective norm) significantly increased workers’ intention to violate and the actual occurrence of violations. Similarly, Wang et al. [40] found that all factors of TPB showed significant effects on violations in lane changing behavior. 

The other type of aberrant behavior, errors, seem to fall outside of TPB’s explanatory scope given that they are unintentional or unplanned behavior. However, some factors of TPB have been reported as significant predictors of errors in safety literature. For instance, Victoir et al. [41] found that perceived behavioral control was a dominant determinant that explained 33% of variance in driving errors. Lucidi et al. [42] and Mallia et al. [43] found that drivers possessing more a positive safety attitude reported fewer errors, suggesting that attitude towards traffic safety rules was a significant predictor of driving errors. Paletz et al. [44] proposed taking subjective norm into consideration when analyzing errors. Based on the above studies, it was hypothesized that TPB should have some explanatory power in regard to errors. 

Finally, although the motivating mechanism of safety participation has not been intensively investigated, there has been evidence suggesting that it falls under the explanatory power of TPB. Fugas et al. [39] applied TPB as the framework to predict safety participation behavior at a transportation organization. They found that subjective norm was the most significant predictor of safety participation behavior of transportation organization workers, followed by safety attitude. However, in their study, perceived behavioral control was not identified as a significant determinant. A more recent study [45] investigating safety participation behavior at construction sites showed that all three factors of TPB were significant predictors.

Taking all evidence together, this study applied TPB as the theoretical framework to predict errors, violations, and safety participation behavior of commissioning workers. It should be noted though, that when predicting a specific type of behavior, most TPB-related studies have measured the perception or attitude towards this specific behavior. For instance, attitudes towards violations were measured when using TPB to predict violations [12]. However, instead of measuring the specific perception towards each type of behavior, this study used a general perception towards safety regulations to predict the three types of behavior. That is, we have measured the attitude, subjective norm, and perceived behavioral control towards safety regulations.

Based on the above evidence, it was hypothesized that:

**Hypothesis 1** **(H1).** *Attitude would have a direct negative effect on errors and violations and a direct positive effect on safety participation.*

**Hypothesis 2** **(H2).** *Subjective norm would have a direct negative effect on errors and violations and a direct positive effect on safety participation.*

**Hypothesis 3** **(H3).** *Perceived behavioral control would have a direct negative effect on errors and violations and a direct positive effect on safety participation.*

With regard to the relations among the three predictors, TPB assumes that they are correlated. However, many empirical studies have demonstrated that subjective norm and perceived behavioral control are predictors of attitude [12,35]; therefore, it was further hypothesized that:

**Hypothesis 4** **(H4).** *Subjective norm would have a direct positive effect on attitude.*

**Hypothesis 5** **(H5).** *Perceived behavioral control would have a positive effect attitude.*

### 1.3. Executive Function

TPB offers theoretical explanations in terms of why certain behavior would be planned and whether the planned behavior would be carried out or not. However, whether a behavior can be executed the way it is planned also depends on individuals’ executive ability. For instance, there are situations where individuals intend to comply with rules but end up failing to do so due to a low level of executive function [34,46,47,48]. Executive function includes many aspects relevant to successful task completion, such as organization and planning, inhibiting responses, thinking abstractly, and reallocating mental resources [47]. In the context of commissioning, the aspect of executive function most relevant to task completion is probably organization and planning. Organization and planning refer to the ability to think ahead and to carry out organized behavior through functions like multitasking, sequencing, and holding information in mind to make decisions [49]. As mentioned above, commissioning is characterized by collaborations within and across teams, in addition to a tight schedule, all of which require organization and planning ability to coordinate different parties and manage the projects [7]. Some empirical studies found that organization and planning ability could reduce errors associated with ineffective communication or planning and reduce regulation violations due to inappropriate organization of tasks [46]. Although little attention has been paid to the effect of organization and planning ability on safety participation, it was expected that better organization and planning ability could help workers improve work efficiency, therefore giving them more time and energy to engage in voluntary safety promotion programs. Based on the above evidence, it is therefore hypothesized that:

**Hypothesis 6** **(H6).** *Organization and planning would have a positive effect on errors and violations and a negative effect on safety participation.*

### 1.4. Proposed Model

**Figure 1:**
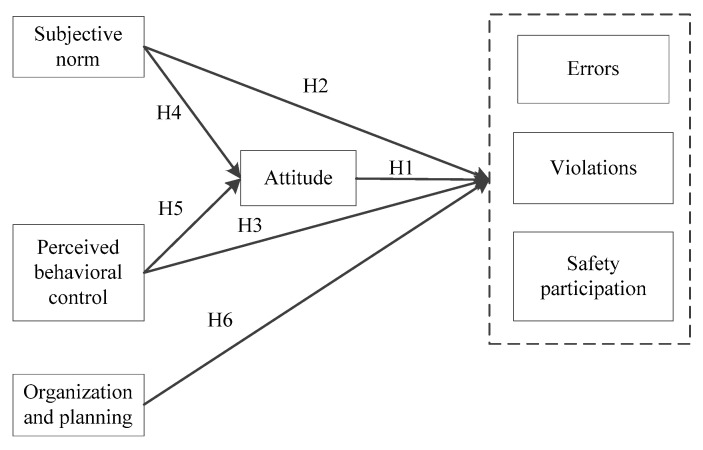
Proposed model for explaining errors, violations, and safety participation behavior of commissioning workers.

The proposed model is shown in Figure 1. Utilizing the TPB framework, it is hypothesized that attitude, perceived behavioral control, and subjective norm would directly impact the occurrence of errors, violations, and safety participation behavior. Besides, subjective norm and perceived behavioral control could indirectly affect workers’ behavior through the attitude factor. Moreover, organization and planning ability is a direct determinant of commissioning workers’ behavior.

## 2. Materials and Methods

### 2.1. Questionnaire

Data used in this study were extracted from a large questionnaire survey investigating the relationship between personality, cognitive abilities, and performance of commissioning workers. The questionnaire was designed after an extensive review of the literature and revised based on the feedback from three commissioning experts to improve its clarity and readability. Three experts on human factors were also consulted to guarantee the quality of the questionnaire. Questions related to this study are introduced below and listed in detail in Table A1 in Appendix A.

Basic information inventory: This part was designed to collect demographic information including age, gender, educational level, marriage status, working experience, and working division (i.e., technique management, electrical commissioning, conventional island commissioning, instrumentation and control commissioning, nuclear island commissioning, or other).

Behavior: This part required respondents to rate their frequency of errors, violations, and active safety participation behavior at work on a 5-point Likert scale ranging from 1 = “never” to 5 = “always”. Seven items from Rao et al. [50] were adopted to measure errors (e.g., “I promised to return to someone with information but forgot to do so.”), and three items from the same source as above were used to measure violations (e.g., “sometimes I do not use the correct safety procedures for carrying out my job”). Based on its definition and discussion with commissioning experts, three items were developed to measure safety participation (e.g., “I would alert my co-workers if potential safety concerns exist”).

TPB factors: This section was used to collect respondents’ perceptions and attitudes towards safety regulations at work. In particular, subjective norm was measured by adjusting the three items from Venkatesh and Davis [51] (e.g., “people who influence me a lot think I should follow rules and procedures at work”); perceived behavioral control was measured by adapting the five items from Taylor and Todd [52] (e.g., “I am able to follow rules and procedures at work”); and attitude was measured using three items from Iversen [53] (e.g., “many rules must be ignored to ensure work flow”). Items were also rated with a 5-point Likert scale ranging from 1 = “totally disagree” to 5 = “totally agree”.

Organization and planning: This factor was measured using the twelve items from the Executive Function Index (EFI) developed by Spinella [49]. Respondents were required to indicate the extent to which they agreed with descriptions of themselves in daily life (e.g., “organized person”). Similarly, a 5-point Likert scale ranging from 1 = “totally disagree” to 5 = “totally agree” was used by respondents to rate their answers.

### 2.2. Participants

Face-to-face invitation to participate in the survey was given by visiting the commissioning offices at Yangjiang and Taishan nuclear power plants in July of 2019. These two nuclear power plants are located in Guangdong Province in China, and both of them belong to the General Nuclear Power Corporation. Therefore, they are comparable in organizational structures, work regulations, and work environments. The ethics approval is included as parts of the official contract of the collaboration project between Shenzhen University and the State Key Laboratory of Nuclear Power Safety Monitoring Technology and Equipment of China. The ethic approval code is 007-EC-B-2019-C83-P.S.20-01122. Participants were informed that they could quit the survey at any time and all data collected would be handled anonymously and confidentially and only be used for research purposes. A total of 187 questionnaires were distributed, and 179 were returned within the required time period. Of the returned questionnaires, 12 were excluded from further analysis because of the high proportion of unanswered questions (>20%). Data from the remaining 167 questionnaires were used in this study.

Of the valid responses, 130 (77.8%) were from Yangjiang nuclear power plant, and 37 (22.2%) were from Taishan nuclear power plant. Almost all respondents (*n* = 165, 98.8%) were males. On average, they were 32.5 years old (SD = 4.8), and they had an average working experience of 7.7 years (SD = 2.4). The majority of the respondents were married (*n* = 123, 73.6%). With regard to educational level, the majority (*n* = 146, 88.0%) had a bachelor’s degree, a few had a master’s degree (*n* = 13, 7.8%), and the rest had a college degree or below (*n* = 7, 4.2%). Given that the distribution of gender, marriage status, and educational level variables were highly skewed, their possible confounding effects on the proposed model were not investigated. The workers were distributed in different divisions: technique management (*n* = 21, 12.5%), electrical commissioning (*n* = 30, 18.0%), conventional island commissioning (*n* = 39, 23.3%), instrumentation and control commissioning (*n* = 36, 21.6%), nuclear island commissioning (*n* = 34, 20.4%), and other (*n* = 7, 4.2%).

### 2.3. Data Analysis

A two-step procedure for conducting structural equation modelling (SEM), as proposed by Anderson and Gerbing [54], was adopted. In the first step, a series of tests were carried out to evaluate the reliability and validity of the measurement model. In particular, internal consistency was evaluated with Cronbach’s α. A value of Cronbach’s α greater than 0.6 indicates acceptable internal consistency [55]. Confirmatory factor analysis (CFA) was used to examine whether multiple items of the same factor were in agreement, i.e., the convergent validity. Convergent validity is achieved when the model shows a good fit and the factor loading of an item on its posted underlying factor is significant and larger than 0.5 [56]. The model was considered to have a good fit when the following criteria were fulfilled: ratio of chi-square value to degree of freedom (χ^2^/*df*) < 3, comparative fit index (CFI) ≥ 0.90, Tucker–Lewis index (TLI) ≥ 0.90, incremental fit index (IFI) ≥ 0.90, standardized root-mean-square residual (SRMR) < 0.08, and root-mean-square error of approximation (RMSEA) < 0.06 [57,58,59,60]. Discriminant validity reflects the extent to which the factors differ from one another empirically [56]. According to the Fornell and Larcker criterion, discriminant validity is achieved if the square root of average variance extracted (SAVE) for each of the factors is greater than any of the bivariate correlations involving the factor in the model [61].

In the second step, SEM was used to assess the goodness of fit of the proposed model and to investigate the strength of the relationships among factors in the model (i.e., hypothesis testing). The same goodness-of-fit criteria (i.e., χ^2^/*df* < 3, CFI ≥ 0.90, TLI ≥ 0.90, IFI ≥ 0.90, SRMR < 0.08, and RMSEA < 0.06) were applied to evaluate the fit of the proposed model. All analyses were performed in R software (R Foundation for Statistical Computing, Vienna, Austria) (R Core Team. (2016). R: A Language and Environment for Statistical Computing. Vienna, Austria. Retrieved from http://www.R-project.org/).

## 3. Results

### 3.1. Reliability and Validity of the Measures

The CFA results showed that the factor loadings of five items from the organization and planning factors (marked in the Table A1) were smaller than the required minimum value of 0.5 and therefore were removed from further analysis. The CFA was then reconducted. The revised model showed a satisfactory goodness of fit (χ^2^/*df* = 1.6, CFI = 0.91, TLI = 0.90, IFI = 0.91, SRMR = 0.072, and RMSEA = 0.059; see Table 1). All items in the revised model had factor loadings larger than 0.5, suggesting an acceptable convergent validity of the factors. All Cronbach’s α values were greater than 0.6 (Table 1), suggesting a good internal consistency of these factors. The SAVE (shown on the diagonal in Table 1) of each factor was greater than the associated interfactor correlations (shown off the diagonal in Table 2), indicating acceptable discriminant validity. In addition, all the interfactor correlations are smaller than 0.85, suggesting that multicollinearity is not a problem [62]. In summary, the measurement model showed a satisfactory reliability and validity and was appropriate for the analysis of the structure model. Furthermore, given that age and working experience showed no significant effects on variables in the proposed model, their effects were not controlled in the following SEM analysis.

### 3.2. Structure Model

**Figure 2:**
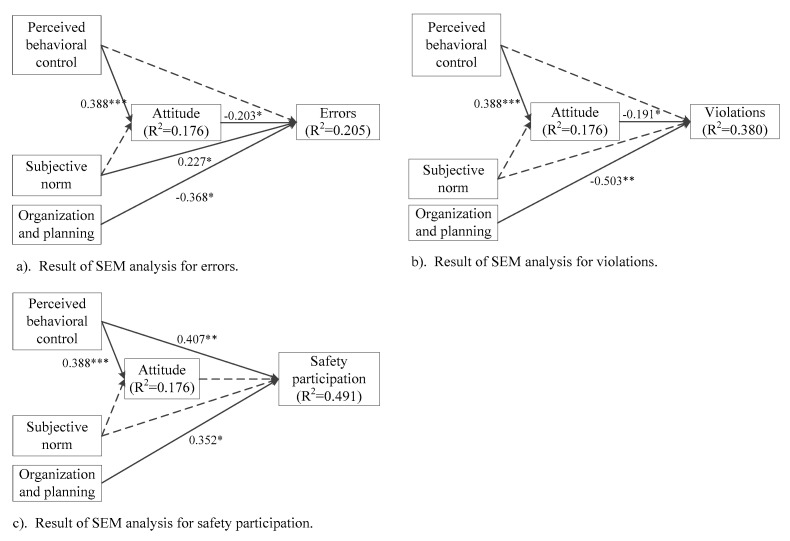
The final model and the significant standardized path coefficients, (**a**) result of SEM analysis for errors, (**b**) result of SEM analysis for violations, (**c**) result of SEM analysis for safety participation. * *p* < 0.05; ** *p* < 0.01; *** *p* < 0.001; dotted line represents a nonsignificant path parameter.

The SEM results suggested that our model had a satisfactory goodness of fit, with χ^2^/*df* = 1.6, CFI = 0.91, TLI = 0.90, IFI = 0.91, SRMR = 0.072, and RMSEA = 0.059. The estimated standardized path coefficients of the significant relationships in the model and the proportion of explained variance (R^2^) are shown in Figure 2. For clarity, models of the three kinds of behavior (i.e., errors, violations, and safety participation) are presented separately. For relationships within the three factors from TPB, attitude was significantly predicted by perceived behavioral control (β = 0.338, *p* < 0.001), with a higher level of perceived behavioral control over following safety rules leading to better attitude. The effect of subjective norm on attitude did not reach a significant level.

Of the three types of behavior, errors were directly determined by attitude (β = −0.203, *p* = 0.036), subjective norm (β = 0.227, *p* = 0.009), and organization and planning (β = −0.368, *p* = 0.012). As hypothesized, workers with better attitude and a higher organization and planning ability had fewer errors. Perceived behavioral control did not have a significant direct effect on errors (β = 0.042, *p* = 0.736). However, it showed a significant effect on attitude, which further significantly predicted errors. Therefore, perceived behavioral control could indirectly influence the occurrence of errors by affecting attitude. Unexpectedly, workers who perceived a higher norm that they should follow rules and procedures were more likely to make errors. A total of 20.5% of the variance in errors was explained by the proposed model.

With regard to violations, the path coefficients of attitude (β = −0.191, *p* = 0.036) and organization and planning (β = −0.503, *p* = 0.001) were significant. Again, perceived behavioral control did not show a direct effect on violations (β = −0.049, *p* = 0.694) and could only indirectly influence it through attitude. Subjective norm was not a significant predictor of violations either (β = −0.005, *p* = 0.953). A total of 38.0% of the variance in violations was explained.

For safety participation, the most significant predictor was perceived behavioral control (β = 0.407, *p* = 0.002), followed by organization and planning (β = 0.352, *p* = 0.012). These two factors explained as high as 49.1% of the variance in safety participation. Attitude (β = 0.055, *p* = 0.549) and subjective norm (β = −0.093, *p* = 0.252) did not show any significant effects in shaping workers’ safety participation behavior. Table 3 summarizes the results of the hypothesis tests.

## 4. Discussion

Commissioning workers have long been ignored in previous studies although their performance is closely related to the overall safety of nuclear power plants. This study aimed to investigate the determinants of three types of behavior (i.e., errors, violations, and safety participation) of commissioning workers, under the general framework of TPB and by considering organization and planning factors. Our work demonstrated the usefulness of TPB in explaining commissioning workers’ behavior and revealed the working mechanisms under the three types of investigated behavior.

All three types of behavior were significantly predicted by workers’ organization and planning ability. First, commissioning workers with a better organization and planning ability reported fewer errors. This is probably because those good at multitasking, sequencing, and planning are better prepared to deal with the pressure of time and the challenge of technological complexity during commissioning work, which have been reported in literature as major contributors to errors [12,63]. Another possible explanation is that those with a better organization and planning ability are less likely to engage in distracting activities [64] and therefore may commit fewer errors associated with distraction. Second, consistent with findings in the context of driving [46,48], our results show that violations were negatively associated with organization and planning. According to Lund and Rundmo [65], some violations are a result of underestimation of possible harmful consequences associated with such behavior. Organized persons, who carry out careful and comprehensive evaluation before taking action, are more likely to realize the negative consequences of violations and therefore less likely to violate rules. Finally, active safety participation was positively associated with organization and planning ability, probably because those with clear plans for the future would devote greater efforts to activities that are beyond their formal role but can contribute to realizing their future goals, like safety participation activities. Together, our results demonstrate that organization and planning affect all kinds of behavior and are critical to the overall safety of commissioning work.

A favorable attitude towards rules and procedures resulted in fewer errors and violations but did not promote voluntary safety participation behavior. This result agrees with previous evidence that violations are deliberate and planned behavior and such behavior is a direct result of attitude [12,37,40]. Errors, which are supposed to be unintended or unplanned behavior, were also partially determined by attitude. This is probably because those showing a positive attitude towards safety rules and procedures pay more attention to work, which helps reduce the probabilities of temporary failures of concentration, memory, or judgement that may cause errors. Surprisingly, contrary to previous results [39,66], better attitude did not promote safety participation, but better perceived behavioral control and organization and planning did. These results in combination suggest that active involvement in safety promotion depends on organizational and individual support, not on individual preference.

The three types of behavior showed different relationships with subjective norm. On one hand, behavior with clear intention, i.e., violation and safety participation, was not predicted by subjective norm in this study. This finding is inconsistent with previous studies which have found that behavior at workplaces like those of transportation [39,40] and construction [67,68] was related to subjective norm. This unexpected finding is probably a result of the specialization of commissioning work. Commissioning work requires specified knowledge and professional training, and workers might believe that others, especially those not working in commissioning, know very little about their work. Therefore, commissioning workers may not take his family members’ or friends’ opinions into consideration when planning intended behavior, i.e., violations and safety participation. On the other hand, subjective norm positively impacted the occurrence of errors. A possible explanation is that while perceived norm did not impact intended decisions, it is a kind of pressure that might lead to unintentional lapses and slips [69]. 

Finally, perceived behavioral control was a critical factor of commissioning safety, as it affected all three types of behavior. Consistent with much evidence in literature [70], those who perceived themselves as more capable of following rules and procedures at work had a more positive attitude towards rules and further reported fewer errors and violations. More importantly, our results showed that commissioning workers who had higher perceived behavioral control were more likely to actively participate in safety promotion activities. This suggests that, besides the widely recognized factors such as supervisor leadership [71,72] and safety climate [73,74], perceived behavioral control should also be considered when developing interventions to promote workers’ safety participation.

## 5. Conclusions

This study is one of the first studies to investigate the behavior of commissioning workers at nuclear power plants. The main findings include that (1) all three types of investigated behavior (i.e., errors, violations, and safety participation) are significantly predicted by perceived behavioral control and organization and planning; (2) violations and errors are a direct result of attitude towards safety rules and procedures, while safety participation is not related to such attitude; and (3) errors are further positively predicted by subjective norm. These findings revealed the underlying mechanisms for the development of errors, violations, and safety participation among commissioning workers and provided practical implications for safety improvement at the commissioning workplace.

Findings of this study have several practical implications for safety improvement at the commissioning workplace. Given the important and positive role of organization and planning in shaping commissioning workers’ behavior, it is recommended that organization and planning ability could be used as a reference when hiring commissioning workers or be considered as content for training in worker improvement programs. In addition, ways to improve workers’ feelings of being in control of their behavior, such as making safety equipment easier to use or offering enough personnel trainings, are of great importance for reducing violations and errors and promoting safety participation. Another remediation of errors is to reduce the level of perceived subjective norm, given that this factor was identified as a significant positive predictor of errors. Practically, this can be achieved by avoiding the emphasis of work regulations by friends and family members.

## Figures and Tables

**Table 1:** Fit indices for the tested models.

Fit Indices	Recommended Values	CFA	SEM
χ^2^/*df*	<3	1.6	1.6
CFI	≥0.90	0.91	0.91
TLI	≥0.90	0.90	0.90
IFI	≥0.90	0.91	0.91
SRMR	<0.08	0.72	0.072
RMSEA	<0.06	0.059	0.059

**Table 2:** Mean, standard deviation (SD), and zero-order correlation between factors.

Variables	1	2	3	4	5	6	7	8	9
1. Age	−								
2. Working Experience	0.58 ***	−							
3. Organization and Planning	0.08	0.12	(0.71)						
4. Subjective Norm	−0.24 **	−0.11	0.04	(0.92)					
5. Perceived Behavioral Control	0.09	0.20	0.42 ***	0.06	(0.80)				
6. Attitude	0.12	0.12	0.36 ***	0.14	0.62 ***	(0.61)			
7. Errors	−0.08	0.03	−0.41 ***	0.13	−0.25 ***	−0.31 ***	(0.68)		
8. Violations	−0.10	−0.13	−0.38 ***	−0.10	−0.37 **	−0.42 ***	0.26 **	(0.71)	
9. Safety Participation	0.07	0.13	0.36 ***	−0.01	0.45 ***	0.58 ***	−0.31 ***	−0.42 ***	(0.61)
Mean	32.50	7.73	3.64	3.49	4.12	4.03	2.22	1.95	3.76
SD	4.75	2.36	0.39	0.89	0.45	0.47	0.49	0.60	0.54
Cronbach’s α	−	−	0.78	0.94	0.90	0.80	0.83	0.76	0.67

Note: The numbers in parentheses are the SAVEs. The off-diagonal elements are the correlations between the factors. ** *p* < 0.01; *** *p* < 0.001.

**Table 3:** Summary of the hypothesis test results.

Hypotheses	Paths	Supported?
H1	Attitude → Behavior	Supported in Errors and Violations; Rejected in Safety Participation;
H2	Subjective norm → Behavior	Supported in Errors; Rejected in Violations and Safety Participation;
H3	Perceived Behavioral Control → Attitude	Supported in Safety Participation; Rejected in Errors and Violations;
H4	Subjective Norm → Behavior	Rejected
H5	Perceived Behavioral Control → Attitude	Supported
H6	Organization and Planning → Behavior	Supported

## Appendix A

**Table A1:** List of items used in this study.

Factors	Items	References
Subjective norm	People who influence me a lot think I should follow rules and procedures at work.	[51]
	People who are important to me think I should follow rules and procedures at work.	
	My colleagues think I should follow rules and procedures at work.	
Perceived behavioral control	I am able to follow rules and procedures at work.	
	Following rules and procedures at work is under my control.	
	I am capable of following rules and procedures at work.	
	I have resources to follow rules and procedures at work.	
	I have the knowledge to follow rules and procedures at work.	
Attitude	Many rules must be ignored to ensure work flow. (*)	[53]
	Some procedures can be ignored because they are too restrictive. (*)	
	Work rules are often too complicated to be carried out in practice. (*)	
Organization and planning	Organized person	[49]
	Save money regularly (^#^)	
	Self-monitor for mistakes	
	Plan for the future (^#^)	
	Use of memory strategies (^#^)	
	Anticipate consequences of actions (*)	
	Learn from mistakes	
	Trouble summing information for decisions (^#^)(*)	
	Distractibility (*)	
	Lost track of what I am doing (*)	
	Mix up the sequences of actions (*)	
	Trouble doing two things at once (^#^)(*)	
Error	Promised to return to someone with information but forgot to do so	[50]
	Forgot to perform an operation in a sequence of operations that I planned to carry through	
	Was forced to interrupt a surveillance and control task because I was disturbed by someone and forgot to return to what I was busy doing	
	I misinterpreted a situation and therefore made an error.	
	I was not attentive enough and therefore I missed important information.	
	I could not remember essential information while performing an operation and therefore I had to ask for information again.	
Violation	Sometimes I do not use all the necessary safety equipment to do my job.	[50]
	Sometimes I do not use the correct safety procedures for carrying out my job.	
	Sometimes I do not ensure the highest levels of safety when I carry out my job.	
Safety participation behavior	I would alert my co-workers if potential safety concerns exist.	Self-developed
	I would alert my co-workers if they do not follow rules and procedures at work.	
	I would propose suggestions to improve work safety to my supervisor.	

*: Items reverse coded; ^#^: Items excluded from the final model.
